# Supplementary material for: Molecular Characterization of Wheat Stripe Rust Pathogen (Puccinia striiformis f. sp. tritici) Collections from Nine Countries
Source: Int J Mol Sci. 2021 Aug 31;22(17):9457. doi: 10.3390/ijms22179457 (PMC8430876; doi:10.3390/ijms22179457)
Supplement: Supplementary file 1 [file ijms-22-09457-s001.zip › SupFigs.pptx]

## Slide 1
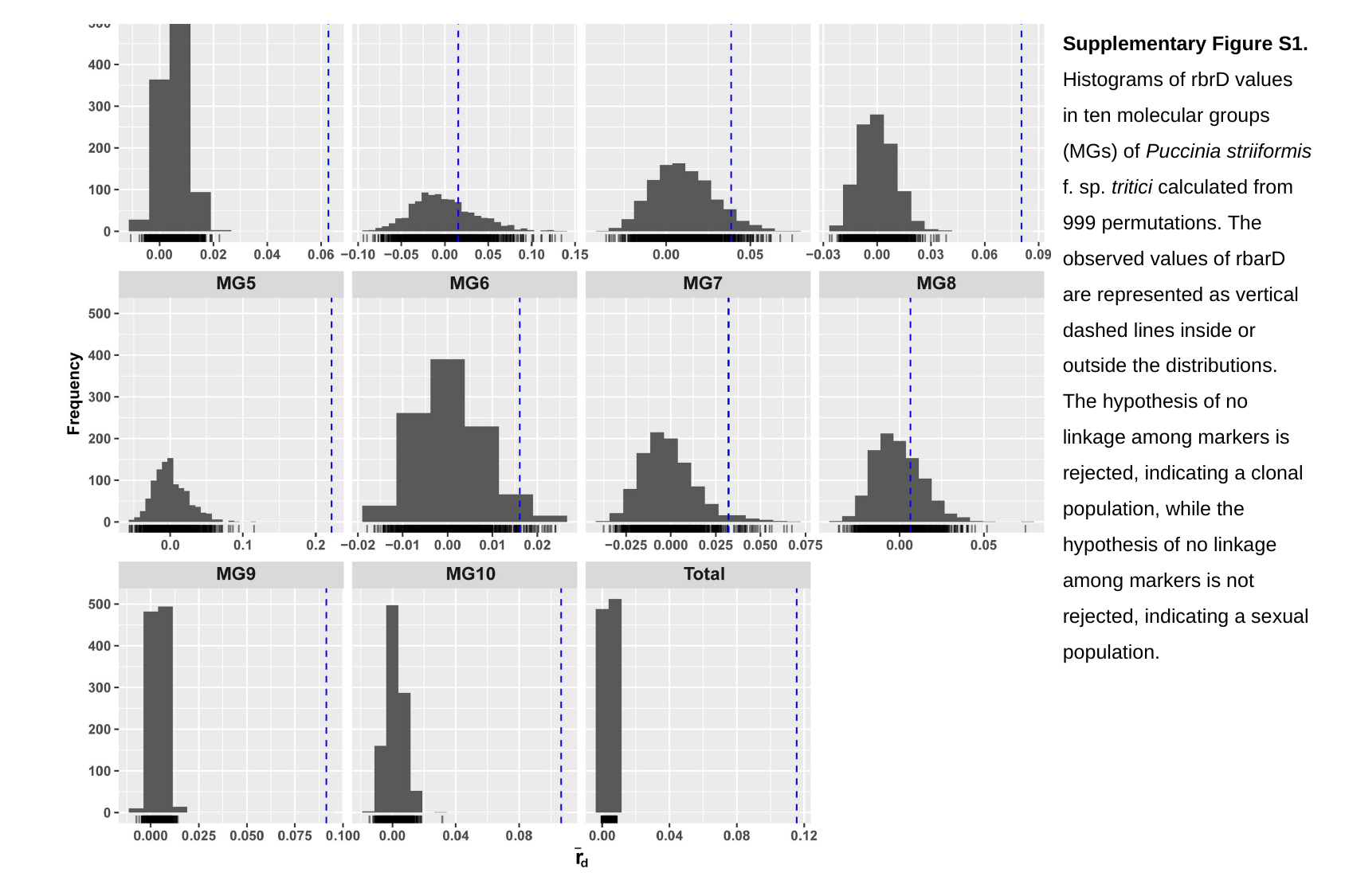

Supplementary Figure S1. Histograms of rbrD values in ten molecular groups (MGs) of Puccinia striiformis f. sp. tritici calculated from 999 permutations. The observed values of rbarD are represented as vertical dashed lines inside or outside the distributions. The hypothesis of no linkage among markers is rejected, indicating a clonal population, while the hypothesis of no linkage among markers is not rejected, indicating a sexual population.

## Slide 2
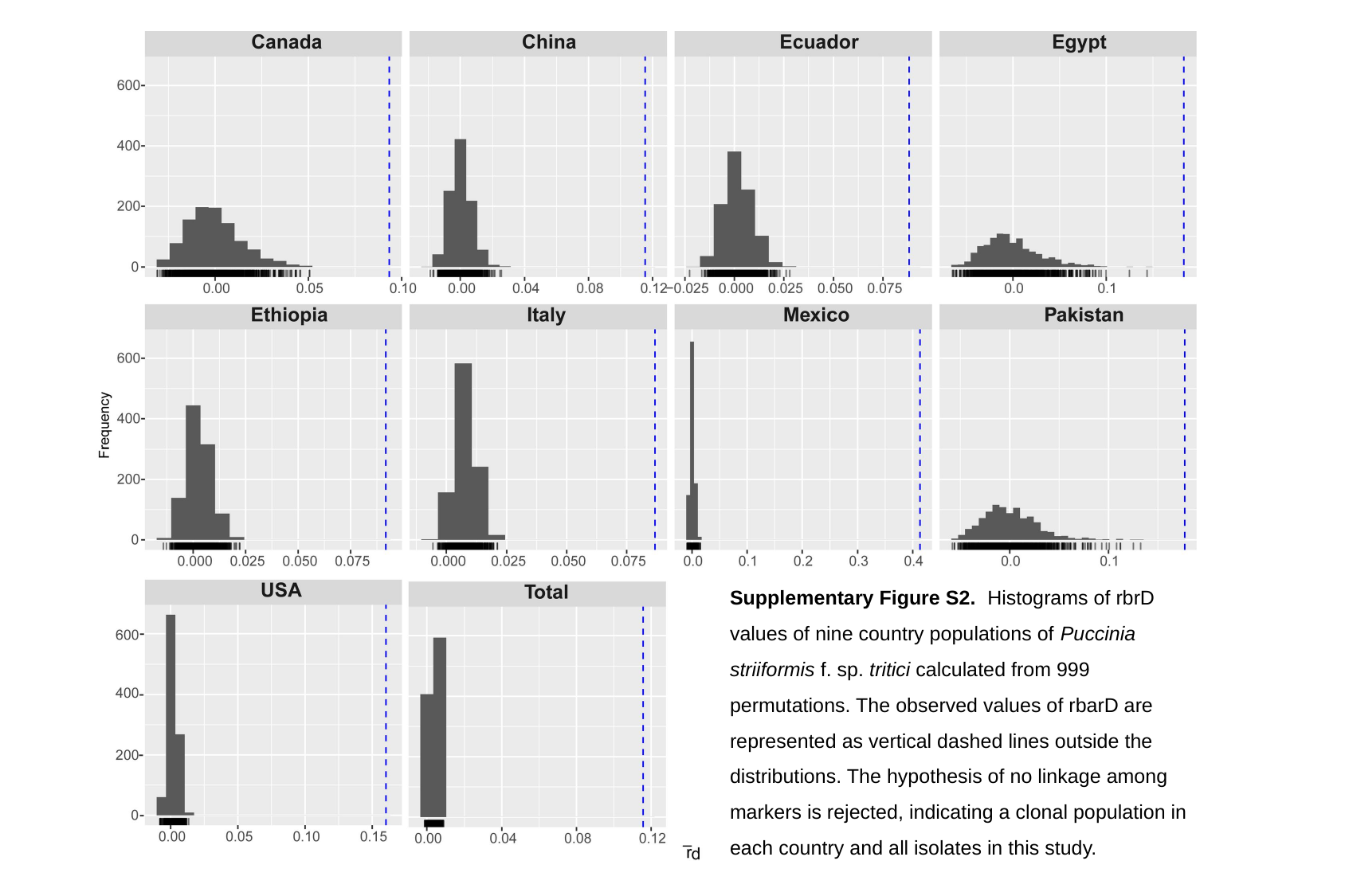

Supplementary Figure S2. Histograms of rbrD values of nine country populations of Puccinia striiformis f. sp. tritici calculated from 999 permutations. The observed values of rbarD are represented as vertical dashed lines outside the distributions. The hypothesis of no linkage among markers is rejected, indicating a clonal population in each country and all isolates in this study.

## Slide 3
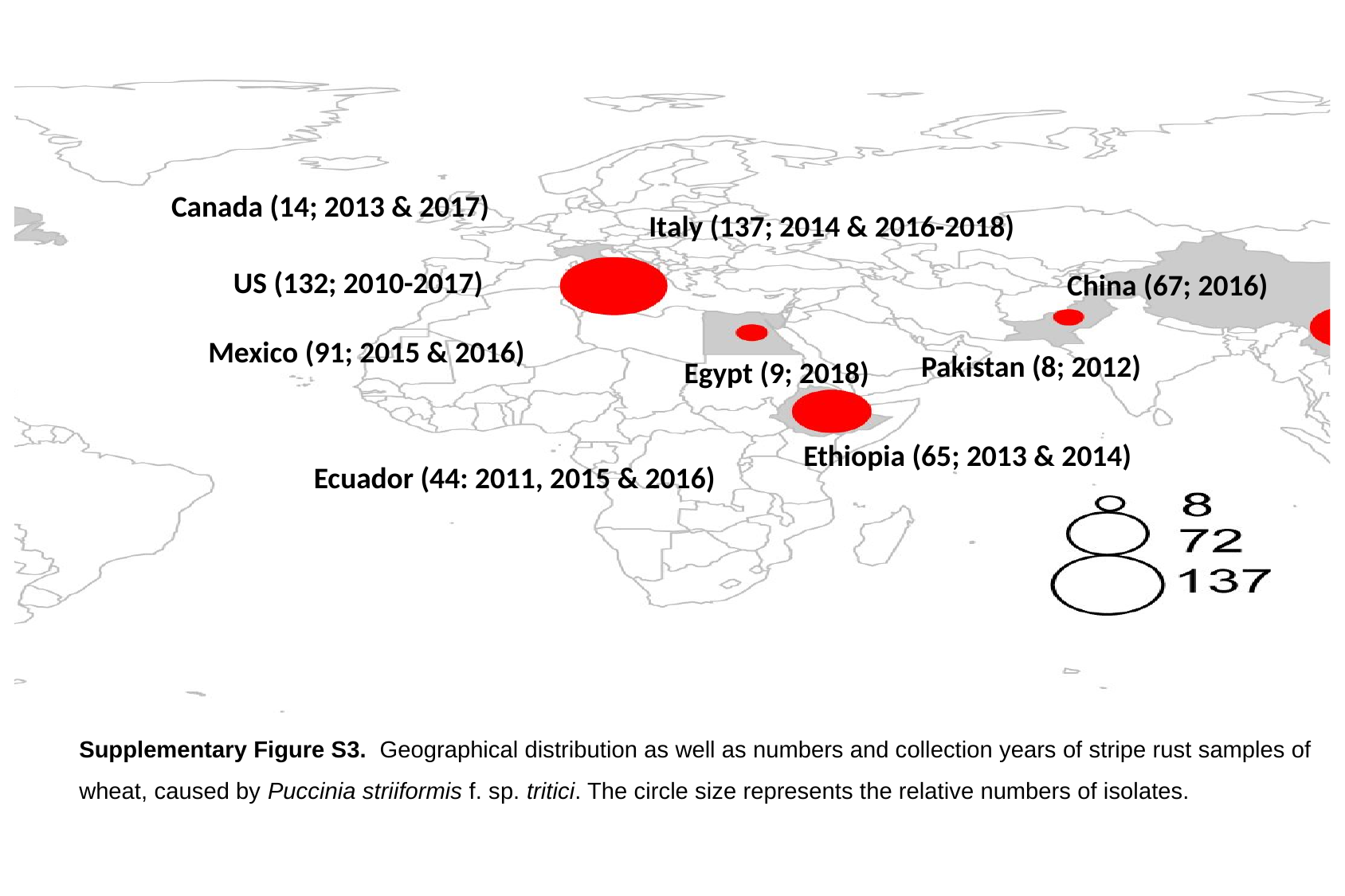

Canada (14; 2013 & 2017)
Italy (137; 2014 & 2016-2018)
US (132; 2010-2017)
China (67; 2016)
Mexico (91; 2015 & 2016)
Pakistan (8; 2012)
Egypt (9; 2018)
Ethiopia (65; 2013 & 2014)
Ecuador (44: 2011, 2015 & 2016)
Supplementary Figure S3. Geographical distribution as well as numbers and collection years of stripe rust samples of wheat, caused by Puccinia striiformis f. sp. tritici. The circle size represents the relative numbers of isolates.
